# Glutathione may have implications in the design of 3-bromopyruvate treatment protocols for both fungal and algal infections as well as multiple myeloma

**DOI:** 10.18632/oncotarget.11592

**Published:** 2016-08-25

**Authors:** Katarzyna Niedźwiecka, Mariusz Dyląg, Daria Augustyniak, Grażyna Majkowska-Skrobek, Magdalena Cal-Bąkowska, Young H. Ko, Peter L. Pedersen, Andre Goffeau, Stanisław Ułaszewski

**Affiliations:** ^1^ Department of Genetics,Institute of Genetics and Microbiology, University of Wroclaw, Wroclaw, Poland; ^2^ Department of Pathogen Biology and Immunology, Institute of Genetics and Microbiology, University of Wroclaw, Wroclaw, Poland; ^3^ KoDiscovery, LLC, UM BioPark, Baltimore, MD, USA; ^4^ Departments of Biological Chemistry and Oncology and Sidney Kimmel Comprehensive Cancer Center (member at large), Johns Hopkins University School of Medicine, Baltimore, MD, USA; ^5^ Institut des Sciences de la Vie, Université Catholique de Louvain, Louvain-la-Neuve, Belgium

**Keywords:** 3-bromopyruvate (3BP), glutathione, buthionine sulfoximine, genes expression, fungi, MM cells

## Abstract

In different fungal and algal species, the intracellular concentration of reduced glutathione (GSH) correlates closely with their susceptibility to killing by the small molecule alkylating agent 3-bromopyruvate (3BP). Additionally, in the case of *Cryptococcus neoformans* cells 3BP exhibits a synergistic effect with buthionine sulfoximine (BSO), a known GSH depletion agent. This effect was observed when 3BP and BSO were used together at concentrations respectively of 4-5 and almost 8 times lower than their Minimal Inhibitory Concentration (MIC). Finally, at different concentrations of 3BP (equal to the half-MIC, MIC and double-MIC in a case of fungi, 1 mM and 2.5 mM for microalgae and 25, 50, 100 μM for human multiple myeloma (MM) cells), a significant decrease in GSH concentration is observed inside microorganisms as well as tumor cells. In contrast to the GSH concentration decrease, the presence of 3BP at concentrations corresponding to sub-MIC values or half maximal inhibitory concentration (IC_50_) clearly results in increasing the expression of genes encoding enzymes involved in the synthesis of GSH in *Cryptococcus neoformans* and MM cells. Moreover, as shown for the first time in the MM cell model, the drastic decrease in the ATP level and GSH concentration and the increase in the amount of ROS caused by 3BP ultimately results in cell death.

## INTRODUCTION

3-bromopyruvate (3BP) is a highly promising anticancer and antifungal drug [1, 2]. Its activity is primarily based on the inhibition of pivotal enzymes of glycolysis, e.g., hexokinase-2, glyceraldehyde-3-phosphate dehydrogenase (GAPDH) [3, 4], and lactate dehydrogenase [4, 5]. Based on the fact that most types of cancer cells exhibit a Warburg effect, i.e., high glycolysis even in the presence of oxygen [6], such cells constitute an ideal target for 3BP, a potent inhibitor of both glycolysis and any remaining mitochondrial oxidative phosphorylation. This leads to rapid death of these cells [4, 5]. Due to the structural similarity to key cellular metabolites, i.e., lactate and pyruvate, 3BP may enter tumor or fungal cells using lactate/pyruvate transporters [7–9]. In contrast, as shown in numerous studies, 3BP's cytotoxicity to healthy mammalian cells is minimal or non-existent [10]. In addition, 3BP inhibits tumor angiogenesis by inhibiting the formation of vascular branching points, reducing the length of vascular tubes, and inhibiting vascular tubulogenesis [11].

Among the changes induced by 3BP at the cellular level, one of the most significant shown in this study is a decrease in the concentration of reduced glutathione (GSH). GSH is a tripeptide thiol consisting of glutamate, cysteine and glycine and is commonly found in eukaryotic and prokaryotic cells. GSH contains a very reactive thiol group (-SH) which makes it a major cellular antioxidant [12]. In addition, it seems that GSH is involved in the repair of DNA as the accumulation of DNA damage was observed in organs of mice that exhibit a defect in GSH metabolism that causes a decrease in the concentration of GSH [13]. High levels of GSH in tumor cells provide significant protection against reactive oxygen species (ROS) thus effectively keeping such cells from undergoing apoptosis [14] and therefore remaining immortal.

In this paper we have examined the phenomenon of apoptosis and necrosis induction by 3BP in the context of GSH and ROS. We have noted that various types of eukaryotic cells are characterized by different natural levels of intracellular GSH. A similar situation was previously described for different species of fungi [15]. Also, our previous studies showed 3BP to exhibit species differentiated antifungal properties. In addition, a significant correlation was found between MIC values for 3BP and the normal cellular concentration of GSH [15]. Therefore, the efficiency of 3BP is clearly dependent on the intracellular level of GSH, and it is not surprising that this agent exhibits a synergistic effect with the known GSH depletors including such compounds as buthionine sulphoximine (BSO), methionine sulfoximine (MSO) and paracetamol (acetaminophen). It is well known for mammalian cells that all of these compounds act as GSH depletors [16–18]. Both BSO and MSO are irreversible inhibitors of γ-glutamylcysteine synthetase and glutamine synthetase respectively, thus reducing the intracellular GSH level [16, 17], whereas paracetamol is metabolized to cytotoxic N-acetyl-4-benzoquinoneimine (NAPQI) that binds directly to the GSH [18, 19]. In this paper we have also shown that 3BP and BSO exhibit a very strong synergistic effect in *Cryptococcus neoformans* cells. Moreover, the stimulating effect of 3BP on the higher expression of genes involved in GSH metabolism has not been previously tested. Although, the important role of GSH is not questionable, it should be noted as previously described that differences in 3BP transport into cells of species differentiated fungi are related directly to observed differences in their susceptibility to this compound [15].

## RESULTS

**Figure 1:**
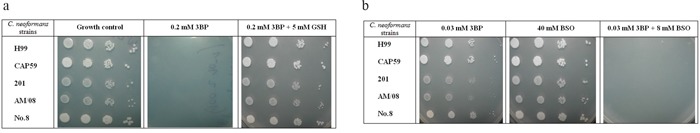
**a, b.** Growth after 72 hrs of incubation at 28°C of clinical and laboratory strains of *Cryptococcus neoformans* on minimal SD medium in the presence of 3BP, GSH and BSO used solely or in combination.

The results of susceptibility tests performed on different fungal cells and micro-algal strains used as models are presented in Table 1. We have observed very different susceptibilities toward 3BP with Minimal Inhibitory Concentration (MIC) values ranging from 0.15 to 8.4 mM. As reported previously, the highest susceptibility toward this compound was shown for *Cryptococcus neoformans* and the lowest for *Exophiala dermatitidis* [15]. Moreover, as predicted synthetic GSH directly added to the SD medium abolished 3BP's inhibitory capacity. In the presence of 5 mM GSH (reduced) in SD medium containing 3BP at a concentration equal to the MIC value (0.2 mM) for the most resistant strain of *Cryptococcus neoformans*, the growth of all tested *Cryptococcus neoformans* strains was restored to that observed for the growth control (Figure 1a).

**Table 1:** Susceptibility of fungal and micro-algal strains used in this study toward 3BP evaluated by the spot-test method and the indicated concentrations of endogenous intracellular GSH

Tested strains	MIC of 3BP [mM]	GSH [μmol/10^6^cells]
*Cryptococcus neoformans* H99	0.15	0.4 ± 0.052
*Cryptococcus gattii* R265	0.6	0.48 ± 0.035
*Cryptococcus uniguttulatus* MD1	2.4	0.85 ± 0.109
*Exophiala dermatitidis* MD1	8.4	1.04 ± 0.052
*Prototheca zopfii*	2.5	2.05 ± 0.40
*Prototheca wickerhamii*	3.0	2.89 ± 0.13
*Prototheca blaschkeae*	3.5	4.86 ± 0.22
*Prototheca zopfii var. hydrocarbonea*	3.5	5.73 ± 0.61

In addition to the just noted results, it was shown also that the combination of low concentrations (significantly below the MIC) of 3BP and BSO completely inhibited growth of the tested *Cryptococcus neoformans* strains (Figure 1b). This effect was apparent when 3BP and BSO were used in combination at concentrations respectively of 4-5 and almost 8 times lower than their MICs (Table 2). Of the five *Cryptococcus neoformans* strains used in this study, the MIC of 3BP was lowered to values equal to 0.03 - 0.04 mM when the SD medium simultaneously contained BSO at a concentration between 8-10 mM. These results clearly indicate a synergistic interaction between 3BP and BSO.

**Table 2:** The MIC and FICI values determined for 3BP and BSO (used solely or in combination) in the case of tested *Cryptococcus neoformans* cells^*^

Compounds	MIC^#^ alone	MIC combined	FICI^##^
3BP	0.15 − 0.2	0.03 − 0.04	0.33 − 0.38 [S]
BSO	60 − 80	8 − 10	

# MIC - Minimal Inhibitory Concentration given at mM of active compound;

## FICI – Fractional Inhibitory Concentration Indices;

* Tests performed on modified minimal synthetic medium (SD) with glucose replaced by sucrose (2%), pH 5.5. Five clinical *Cryptococcus neoformans* strains were included in this study.

**Figure 2:**
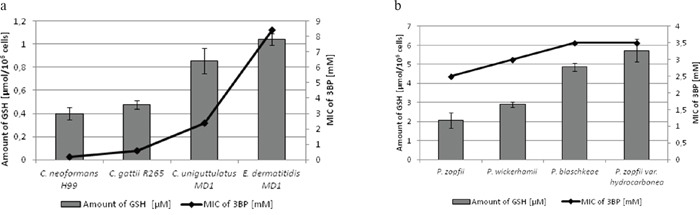
**a, b.** Comparison of the MIC values of 3BP and the concentration of intracellular GSH in fungal (a) and algal (b) cells.

**Figure 3:**
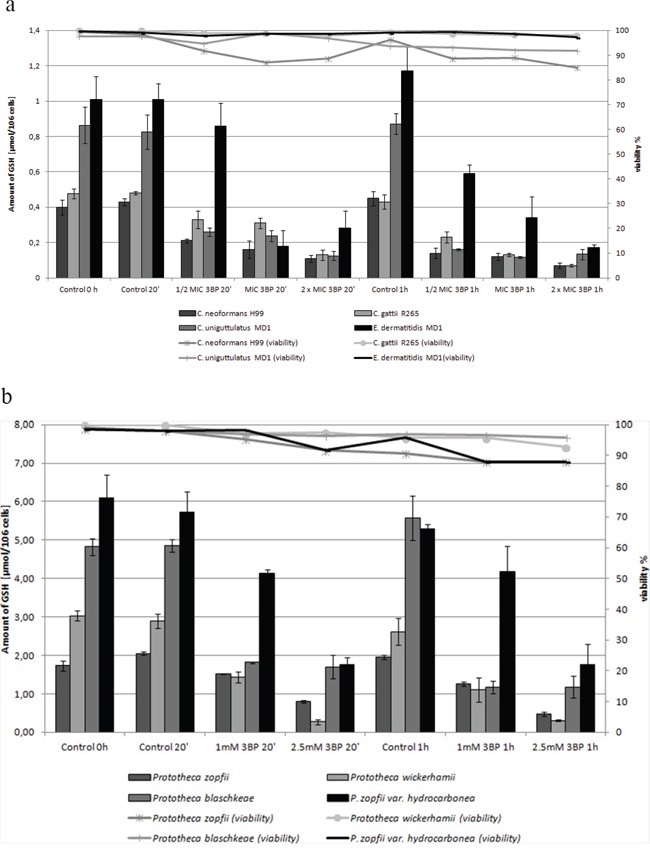
**a.** Influence of 3BP on the viability and GSH concentration inside cells of selected fungi. **b.** Influence of 3BP on the viability and GSH concentration inside *Prototheca spp.* cells.

**Figure 4:**
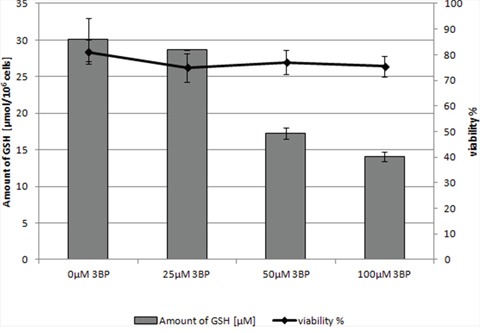
The concentration of intracellular GSH in MM cells (RPMI 8226) after 2 hrs incubation in the presence of 3BP

**Figure 5:**
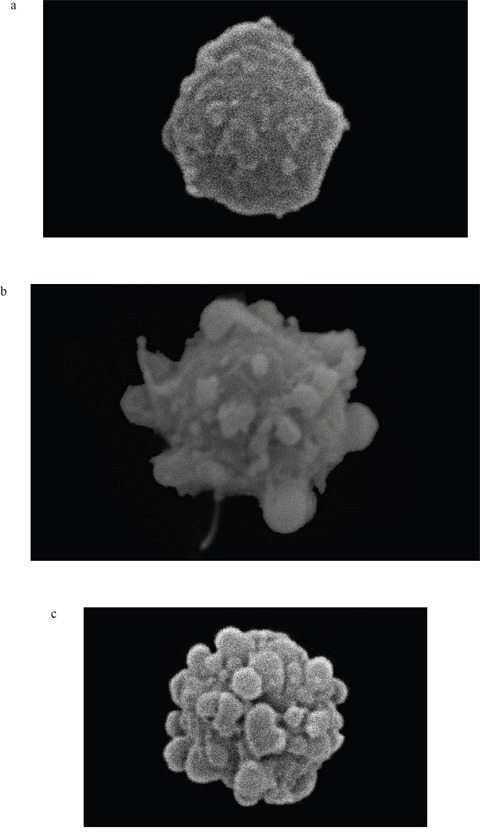
**a-c.** Changes in MM cell morphology under the influence of 3BP (a- 0 μM, b- 50 μM and c- 100 μM) after 2 hrs incubation (SEM, magnification - 5700×).

Moreover, it should be emphasized that we observed a strong correlation between the natural intracellular concentration of GSH and the susceptibility of cells of the analyzed strains toward 3BP. We have demonstrated a high diversity in terms of intracellular levels of the reduced form of glutathione inside cells of species differentiated strains of fungi and microalgae. Both for tested strains of fungi (Figure 2a) as well as algal strains belonging to the Prototheca genus (Figure 2b), we could observe that strains most susceptible to 3BP also have the lowest level of GSH inside their cells (Table 1). In order to examine the effect of 3BP on the intracellular concentration level of GSH, we measured the concentration of this tripeptide in cells of the selected strains which were incubated for a defined time in the presence of appropriate concentrations of this compound. In the case of cells from fungi and algae, we observed after 20 minutes of incubation in the presence of 3BP a significant decrease in GSH concentration. After 1h the concentration remained low or further decreased. During the entire experiment, the viability of cells fluctuated between 80 to 100% depending on the strain and the concentration of 3BP (Figure 3a and 3b). The same observation was made in the case of MM cells. For cell cultures incubated in the presence of various concentrations of 3BP a decrease in GSH concentration was also observed, while their viability was not affected relative to control cells free of this compound (Figure 4). Here, it should be emphasized that during this experiment we observed changes in the morphology of MM cells in the case of samples incubated for 2 hrs in the presence of 3BP at concentrations equal to 50 μM and 100 μM (Figure 5a-5c). No morphological changes were observed in the case of fungal and microalgae cells.

**Figure 6:**
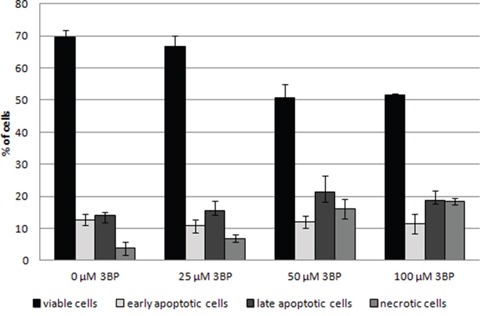
Induction of the apoptosis in MM cells after 2 hrs incubation in the presence of 0 μM, 25 μM, 50 μM and 100 μM 3BP

Observed morphological changes appear to result from apoptosis [20]. In order to determine the cause of morphological changes of MM cells under the influence of 3BP, we have conducted the apoptosis assays. After 2 hrs of incubation in a presence of 25 μM 3BP the percentage of viable, early apoptotic, late apoptotic and necrotic cells was similar to the control sample without 3BP. However, after addition of 50 and 100 μM of 3BP we observed a significant decrease in the number of viable cells, i.e., from ~ 70% to ~ 50% with a simultaneous increase in the percentage of necrotic cells and a slight increase in the number of late apoptotic cells (Figure 6).

**Figure 7:**
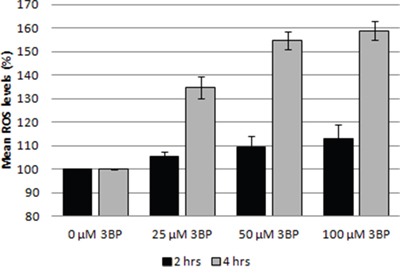
Influence of 3BP (at concentrations of 25 μM, 50 μM and 100 μM) on the generation of ROS in MM cells after 2 and 4 hrs incubation

3BP also influences the generation of reactive oxygen species (ROS) in MM cells. We have clearly observed an increase in the reactive oxygen concentration even at the lowest concentration used for 3BP (25 μM) after 4 hrs incubation. In this case the amount of ROS increased by ~35% compared to the control without 3BP. After 4 hrs incubation in the presence of 50 and 100 μM 3BP the increase was ~55% and ~59% respectively. In contrast, after 2 hrs incubation, changes in the concentration of ROS were much less. Thus, concentrations of 3BP equal to 25, 50 and 100 μM caused approximately 5%, 9% and 13% increase in ROS level, respectively (Figure 7).

**Figure 8:**
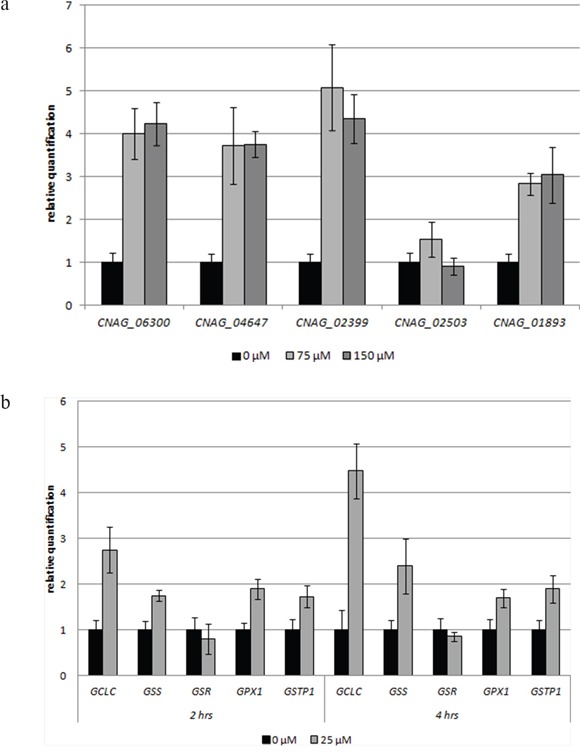
**a, b.** Impact of 3BP on the level of expression of selected genes involved in the metabolism of glutathione in *Cryptococcus neoformans* H99 cells after 4 hrs incubation (a), MM cells after 2 and 4 hrs incubation (b) (*GCLC* and *CNAG_06300* encode γ-glutamylcysteine synthetase, *GSS* and *CNAG_04647*-glutathione synthetase, *GSR* and *CNAG_02399*-glutathione reductase, *GPX1* and *CNAG_02503*-glutathione peroxidase, *GSTP1* and *CNAG_01893*-glutathione S-transferase).

The important role of GSH in the susceptibility of MM cells to 3BP is also clearly visible when the expression level of the genes involved in the metabolism of GSH under the influence of 3BP are analyzed. In comparing the levels of expression of genes encoding γ-glutamylcysteine synthetase (*GCLC* and *CNAG_06300*) and glutathione synthetase (*GSS*, *CNAG_04647)* incubated in the presence of various concentrations of 3BP (25 μM for MM cells and 75 μM or 150 μM for *Cryptococcus neoformans*), we observed a significant increase in both MM and *Cryptococcus neoformans* cells. In the case of MM cells we also noted that the expression of the *GCLC* gene was significantly higher after 4 hrs incubation in the presence of 3BP than after 2hrs. Moreover, we have reported that the genes encoding glutathione S-transferase (*GSTP1, CNAG_01893*) in MM and *Cryptococcus neoformans* cells are also overexpressed under the presence of 3BP. In addition, we observed a slight increase in the expression of the gene that encodes glutathione peroxidase in MM cells. However, we did not note this in the case of *Cryptococcus neoformans.* Rather we reported a strong overexpression of the *CNAG_02399* gene encoding glutathione reductase in *Cryptococcus neoformans.* However, the expression level of the *GRS* gene encoding the same enzyme in MM cells was comparable to the control incubated without 3BP (Figure 8a, 8b). Therefore, we can conclude that while on the one hand our studies show that 3BP causes a decrease in the intracellular GSH concentration, on the other hand 3BP causes a significant increase in the expression level of genes encoding the enzymes contributing to increasing the intracellular concentration of GSH.

## DISCUSSION

The susceptibility tests performed for 3BP and BSO alone and in combination on the *Cryptococcus neoformans* cell model allowed us to show significant hyper-synergism between these compounds. The Fractional Inhibitory Concentration Indices (FICI values) were between 0.33 and 0.38, i.e., below the upper limit value (0.5). We know that BSO causes irreversible inhibition of γ-glutamylcysteine synthetase and for this reason effectively blocks GSH synthesis causing a drastic decrease in its intracellular concentration [16]. In our previous studies we observed similar results with MM cells where pretreatment with 100 μM BSO decreased the half maximal inhibitory concentration (IC_50_) for 3BP from approximately 25 μM to 13 μM [21]. Additional confirmation for an important role for GSH in the generation of a phenotype resulting in natural resistance to 3BP in *Cryptococcus neoformans* cells are results of tests performed with the same SD medium supplemented with GSH at a concentration of 5 mM. Thus, we observed about an 8-fold decrease in susceptibility to 3BP. These results allow us to conclude that 3BP in direct combination with GSH manifests an antagonistic effect.

The study performed on MM cells to determine the impact of 3BP on intracellular GSH concentration resulted in an interesting phenomenon. Thus, the specific morphology of MM cells treated with high concentrations of 3BP (50 μM and 100 μM) resulted in an advanced stage of apoptosis. This was the case for approximately 20% of the total number of cells and confirmed by an apoptosis assay. Thus, after 2 hrs exposure to 3BP we observed the well described morphology of apoptotic cells [20]. They appeared highly dehydrated with drastic changes in shape. In addition, we observed cells that were mainly rounding and shrinking with characteristic membrane bubbles as described many times earlier [20, 22, 23]. Also, it was well demonstrated [22], that the apoptotic cells exhibit the typical disintegration of the nuclear membrane and subsequent nuclear fragmentation. This phenomenon is explained by the so-called CAD/ICAD system. CAD is a specific DNase that in most proliferating cells is expressed as an inactive enzyme complexed with ICAD (inhibitor of CAD). Under the influence of 3BP or other agents that induce apoptosis caspase 3 cleaves ICAD and CAD which causes DNA degradation. Chromatin fragments and cellular organelles become surrounded by portions of the plasma membrane resulting in so-called “apoptotic bodies” that may be phagocytosed later by macrophages [20, 23]. Despite the fact that after incubation for 2 hrs with 3BP (50 and 100 μM), the percentage of apoptotic cells only slightly increased is in an accordance with the image morphology. Upon microscopic observation we noted that about 20% of the MM cells exhibited characteristic morphological changes. This observation, taken together with the results from flow cytometry, suggest that the initial stage of apoptosis is in progress. This supposition was confirmed by an apoptosis assay that we made upon incubation with 3BP for 18 hrs (data not shown). Thus, using 100 μM 3BP we observed the following percentage of viable, early apoptotic, late apoptotic and necrotic cells respectively: 1.5%, 13.5%, 83% and 2%, This outcome supports the view that following treatment with 3BP the number of dead cells due to apoptosis increases with time. Similar observations of morphological changes resulting from treatment with 3BP were observed in an earlier study with hepatocellular carcinoma cells [24].

The most important action of 3BP, i.e., that on cancer cells has been described previously. Thus, 3BP has been shown to be as a strong inhibitor of glycolytic and mitochondrial function [25] and thus results in depletion of intracellular ATP [15, 21, 26]. In addition, we have shown that 3BP targets other cellular areas in MM cells, one of which results in induction of oxidative stress. The resultant increase in ROS (reactive oxygen species) following treatment with 3BP has been observed also in hepatoma cells SNU449 and Hep3B [27], glioma cells [11], HL60 cells [28] and in breast cancer cells (MDA-MB-231, MDA-MB-435) [29]. In all these cases, this phenomenon is likely caused by inhibition of the mitochondrial respiratory chain which leads to the accumulation of intracellular ROS.

In contrast to that noted above, the sensitivity of tested fungi and microalgae species toward 3BP was found to be quite different. Among other factors, their susceptibility depends on the concentration of intracellular GSH as shown earlier [15] as well as in the present study. Therefore, it should be clear as to why a higher concentration of the reduced form of GSH inside cells of fungi results in their higher resistance to 3BP. This resistance is likely due to the function of GSH as the main cellular antioxidant [12, 30]. Furthermore, in the presence of 3BP a decrease in GSH levels in cells of all tested fungal and microalgae strains was observed. A significant decrease of reduced GSH concentration with strong inhibition of antioxidant enzymes such as superoxide dismutase and glutathione S-transferase was shown also in a human erythrocyte model [31]. A decrease of intra-cellular GSH levels mainly occurs due to the action of the enzyme glutathione S-transferase. This enzyme is responsible for generating 3BP-GSH conjugates consistent with it often being referred to as a fundamental detoxification enzyme [32]. This reaction occurs in the cytoplasm and as a result is non-toxic for cell conjugates which are transferred to the vacuole [33]. Our experiments showed that the above reaction takes place most likely with no loss of cell viability. This is because the survival of all tested strains was very similar to that of the control.

On the one hand we found that 3BP can bind directly to GSH and on the other that 3BP alters the expression level of genes encoding enzymes involved in the metabolism of this tripeptide thiol. The most significant change was the over expression of genes encoding γ-glutamylcysteine synthetase and glutathione synthetase both in MM and *Cryptococcus neoformans* cells. This phenomenon occurs most likely as a response caused by the action of highly reactive alkylating xenobiotics like 3BP. Such cells need more GSH in order to inactivate the harmful compound and therefore increase the expression of genes encoding the enzymes involved in its synthesis. Consequently, in both organisms we could also observe an increase in the expression of the gene encoding glutathione S-transferase an enzyme that catalyzes the coupling reaction of GSH with nucleophilic and electrophilic compounds [34, 35]. The increased expression of this gene is most likely a response to a higher demand for formation of GSH-xenobiotic complexes to inactivate 3BP and protect the cell. Such a response is rationale and in fact has been noted in the literature. Thus, key enzymes such as glutathione S-transferase, γ-glutamylcysteine synthetase and glutathione synthetase are highly expressed in cells subjected to stress conditions [36].

Although the intracellular concentration of GSH is very important for 3BP's action and may have implications for clinical treatment of fungal infections and cancer, the entry of 3BP via mono-carboxylate transporters seems to be more essential for treating cancers than fungal related diseases. This was demonstrated in earlier studies by using various fungi [8, 15] and erythrocytes [37]. In the latter study it was shown that 3BP uptake depends significantly on pH in the range of 6.0-8.0 [37]. In addition, the affinity for 3BP transport into breast cancer cell lines is higher when the extracellular milieu is acidic suggesting that the uptake of 3BP may be dependent on the proton-motive force [9]. In summary, we can state that it is easier to overcome an internal barrier in the forms of intracellular GSH than overcome the entry of 3BP via MCTs. The last mentioned phenomenon was reported in our earlier studies with *Saccharomyces cerevisiae* cells [8]. Moreover, it was shown that the 3BP molecule is not a substrate for the pleiotropic drug resistance network (PDR) [8], thus excluding this route to cell detoxification. Regarding the clinical implications of these findings one can say that a special diet and/or BSO may be necessary to eliminate a high glutathione concentration in a patient's body in order to facilitate effective treatment with 3BP.

## MATERIALS AND METHODS

### Reagents

All reagents if not otherwise mentioned were purchased from Sigma-Aldrich (Poland) and were of analytical grade.

### Fungal and algal strains, MM cell cultures and growth conditions

All fungal and algal strains used in this study are listed in Table 3. These strains were isolated from either humans or animals and from the environment as well.

**Table 3:** Fungal and micro-algal strains used in this study and their source

Taxonomic position	Strain	Source
**Fungi**	*Cryptococcus neoformans* H99	Heitman^1^ (clinical strain)
	*Cryptococcus neoformans* CAP59	De Cock^2^ (laboratory strain)
	*Cryptococcus neoformans* 201	Nawrot^3^ (clinical strain)
	*Cryptococcus neoformans* AM/08	Dyląg^4^ (clinical strain)
	*Cryptococcus neoformans* No.8	Dyląg^4^ (clinical strain)
	*Cryptococcus gattii* R265	May^5^ (clinical strain)
	*Cryptococcus uniguttulatus* MD1	Dyląg^4^ (environmental strain)
	*Exophiala dermatitidis* MD1	Dyląg^4^ (clinical strain)
**Algae**	*Prototheca zopfii*	Jagielski^6^ (clinical strain)
	*Prototheca wickerhamii*	Jagielski^6^ (clinical strain)
	*Prototheca blaschkeae*	Jagielski^6^ (clinical strain)
	*Prototheca zopfii var. hydrocarbonea*	Jagielski^6^ (clinical strain)

1 Joseph Heitman – Department of Molecular Genetics and Microbiology, Duke University School of Medicine, Durham, USA

2 Hans de Cock – Department of Biology, Microbiology, Institute of Biomembranes, Utrecht University, Utrecht, The Netherlands

3 Urszula Nawrot – Department of Microbiology, Wroclaw Medical University, Chalubinskiego Street 4, Wroclaw, Poland

4 Mariusz Dyląg – Department of Genetics, Institute of Genetics and Microbiology, University of Wroclaw, Przybyszewskiego Street 63/77, Wroclaw, Poland

5 Robin May – School of Biosciences and Institute of Microbiology & Infection, University of Birmingham, Edgbaston, Birmingham, United Kingdom

6 Tomasz Jagielski – Department of Applied Microbiology, Institute of Microbiology, University of Warsaw, Miecznikowa 1 Street, Warsaw, Poland

All tested strains were grown at 28°C in complete YPD medium (2% w/v peptone, 1% w/v yeast extract, pH=5.5) and in synthetic minimal SD medium (0.67% w/v of yeast nitrogen base without amino acids, pH=5.5). Both types of media contained 2% w/v glucose (in case of algal strains) or sucrose (in case of fungi) as a sole carbon source. All the used media were solidified by agar (2% w/v, Difco). The RPMI 8226 (ATCC^®^ CCL155™) cell line (MM.1 human multiple myeloma) was obtained from the Cell Culture Collection of the Institute of Immunology and Experimental Therapy, Polish Academy of Science, Wroclaw, Poland. The cell cultures were propagated in complete medium (RPMI1640 medium supplemented with 2 mmol/l glutamax, 1% w/v antibiotic-anti-mycotic solution, and 10% w/v heat-inactivated fetal bovine serum) at 37°C in a humidified atmosphere with 5% w/v CO_2_.

### Susceptibility testing

The minimal inhibitory concentration (MIC) values of 3BP and BSO and for combination of these compounds were determined according to a standard spot test method [38]. Fungal and micro-algal cells were grown to an exponential phase and then diluted to an OD_600nm_≈0.125 and spotted (5 μl) in ten-fold serial dilutions (10^0^, 10^−1^, 10^−2^, 10^−3^) onto agar plates containing various concentrations of the tested compound. Plates were incubated at 28°C and photographs were made after 72 hrs (after which we could observe good growth on plates designed for growth control). In addition, the impact of reduced glutathione (synthetic GSH directly added to the SD medium) on its susceptibility to 3BP was monitored (Figure 1b). The susceptibility tests were performed using minimal SD medium with 2% w/v sucrose as the sole carbon source. All tested compounds: GSH (synthetic glutathione), BSO (buthionine sulfoximine) and 3BP (3-bromopyruvate) were dissolved in sterile deionized water (milli Q).

The type of interactions between 3BP and BSO were defined based on calculations of the Fractional Inhibitory Concentration Indices (FICI) [39], where (in this case) FICI = (MIC of 3BP in combination with BSO/MIC of 3BP, alone) + (MIC of BSO in combination with 3BP/MIC of BSO alone). Interactions between compounds were defined as [S] hyper-synergism (FICI ≤ 0.5), [A] additive synergism (0.5 < FICI ≤ 1), [N] neutral action (1< FICI ≤ 2) or [An] antagonistic action (2 < FICI).

### Determination of GSH concentration

Aliquots of 25 ml each were obtained from overnight fungal and algal cultures which had reached an exponential phase of growth (OD_600nm_=1.5-1.8). Then, into Erlenmeyer flasks 3BP at the appropriate concentration was added to those intended as positive controls while in other flasks 3BP was omitted (negative controls). All samples were incubated for a suitable time at 28°C with horizontal shaking (200 rpm/min). Extraction of metabolites was carried out based on the method described by Gonzales *et al*. [40]. For this purpose the whole fungal or algal cell cultures (25 ml) were centrifuged. To the resulting pellet was added 5 ml of buffered boiling ethanol (75% ethanol, 70 mM HEPES, pH 7.5). Then, samples were incubated for 3 min at 80°C and for the next 3 min cooled on ice. Subsequently, all the samples were subjected to evaporation in a vacuum dryer for 3 hrs at 45°C. The intracellular concentration of GSH was determined by using Ellman's reagent [41] as previously described [13]. In the case of MM cells, each sample contained ~4.5 × 10^6^ cells. The samples were incubated for 2 hrs at 37°C in the presence of an appropriate concentration of 3BP. The next steps (metabolite extraction, determination of GSH concentration) were identical for both fungi and microalgae. Cell viability was determined by staining with methylene blue (fungi and Prototheca) or trypan blue (MM cells).

### Induction of apoptosis by 3BP

The induction of apoptosis following the action of 3BP was measured using the Dead Cell Apoptosis Kit with Annexin V FITC (AV) and Propidium iodide (PI) (Life Technologies). MM cells at a concentration of 2.5 × 10^5^ cells per milliliter were treated with the appropriate concentration of 3BP (0 μM, 25 μM, 50 μM and 100 μM) and camptothecin (5 μM) as a positive control. Cells were incubated at 37°C under 5% CO_2_ for 2 hrs. Using BD FACSCalibur™, the fluorescence was detected. Cells were classified as follows: viable cells (AV-/PI-), early apoptotic cells (AV+/PI-), late apoptotic cells (AV+/PI+) and necrotic cells (AV-/PI+).

### Determination of the ROS level

Differences in the generation of free radicals (ROS) were investigated using ROS-Glo™ H_2_O_2_ Assay (Promega). MM cells at a final concentration of 2.5 × 10^5^ cells per milliliter of complete medium were plated in a 96-well-flat-bottom white microplate. After 2 and 4 hrs incubation in the presence of 0 μM, 25 μM, 50 μM and 100 μM 3BP, the luminescence which is proportional to the H_2_O_2_ concentration was measured using a Varioskan™ Flash Multimode Reader.

### Microscopic observation

Materials were fixed in 4% glutaraldehyde in phosphate-buffered saline (PBS, pH 7.4) for 8 hrs. Then, the samples were washed for 24 hrs in 0.2 M PBS (pH 7.4) and treated with 2% osmium tetroxide for 2 hrs. Then, materials were rinsed for half an hour with distilled water, and finally dehydrated by successive immersion in a series of ethanol-water solutions (55%, 70%, 80%, 90%, 96%, 3 × 100%), for 15 min each. Subsequently, samples were transferred to cover slips and hexamethyldisilazane was applied and left to dry. In the next step, materials were mounted on a specimen stub and sputter-coated with spectrally pure carbon and silver in a sputter vacuum. The samples were examined in a scanning electron microscope (SEM) Tesla BS-300.

### Genes expression

The expression level of selected genes within the genome of *Cryptococcus neoformans* (H99 reference strain) was examined based on the real-time polymerase chain reaction (real-time PCR). The overnight fungal cell cultures were incubated for 4 hrs in minimal SD medium in the presence of different concentrations of 3BP (0 μM, 75 μM and 150 μM). MM cells were incubated for 2 and 4 hrs in complete medium containing 0 μM and 25 μM 3BP. The total RNA was isolated using the heat/freeze method [42]. The concentration of RNA was determined using a spectrophotometer (Nanophotometer Pearl, IMPLEN). Subsequently, isolated RNA was reverse-transcribed to cDNA using a high-capacity cDNA reverse transcription kit (Applied Biosystems). Gene expression was measured using a DyNAmo HS Green qPCR kit (Thermo Scientific Finnzymes) via a 7500 real-time PCR System (Applied Biosystems). The final volume of 20 μl of each sample contained 0.5 μM of each primer and 2 μl of cDNA. The thermal cycling conditions were as follows: 95°C for 10 min, followed by 40 cycles at 95°C for 15 s, 58°C for 20 s and 72°C for 30 s. The sequences of primers (designed based on the sequence of genes deposited in the GenBank database) are presented in Table 4. After completion of the reaction, the software (Applied Biosystems), based on the Cq value, calculated the relative quantity of the product for each gene. The *CNAG_00483* (actin) and *GAPDH* in MM cells (housekeeping genes) were used for normalization. Results were obtained from three independent experiments in which the total RNA was isolated from the new culture.

**Table 4:** Oligonucleotides used for real-time PCR assays

Primer	Sequence 5′ - 3′
*GAPDH*_For	GGCATGGCCTTCCGTGTCCC
*GAPDH*_Rev	TGCCAGCCCCAGCGTCAAAG
*GSS*_For	GCGGAGGAAAGGCGAACTA
*GSS*_Rev	AGAGCGTGAATGGGGCATAG
*GSR*_For	TGGCACTTGCGTGAATGTTG
*GSR*_Rev	CTCACATAGGCATCCCGCTT
*GCLC*_For	ACTTCATTTCCCAGTACCTTAACA
*GCLC*_Rev	CCGGCTTAGAAGCCCTTGAA
*GPX1*_For	GCGGGGCAAGGTACTACTTA
*GPX1*_Rev	TCTTGGCGTTCTCCTGATGC
*GSTP1*_For	GCCCTACACCGTGGTCTATTT
*GSTP1*_Rev	GGTCTCCGTCCTGGAACTTG
*CNAG_00483*_For	TCTGGTATGTGCAAGGCTGG
*CNAG_00483*_Rev	CGTAAGAGTCCTTCTGGCCC
*CNAG_04647*_For	CCAACGAGAAGGTGGTGGAA
*CNAG_04647*_Rev	TGACGAGCCAGTTCTCCAAC
*CNAG_02399*_For	TGACTTCAACTGGACCGAGC
*CNAG_02399*_Rev	ACGGTGTACTTGTCGCCATT
*CNAG_06300*_For	AGGCTATACCCGACCAGTGT
*CNAG_06300*_Rev	ATGACTGCAGGTACGCACAA
*CNAG_02503*_For	TCGCCCAGTTTTGTACCCTC
*CNAG_02503*_Rev	TGGTAAAGTTCCACTTGATGGC
*CNAG_01893*_For	CGTCACTCACAAGTGCAAGC
*CNAG_01893*_Rev	CTTTTCAAGATGAGCCGCCG

### Statistical analysis

Results are presented as the mean ± SD from of at least three independent experiments. Statistical significance was assessed by one-way analysis of variance (ANOVA) using GraphPad Prism5, and with Tukey's multiple comparison test. The minimal level of significance was *P*=0.05.
